# Clinical supervision and support: Perspectives of undergraduate nursing students on their clinical learning environment in Malawi

**DOI:** 10.4102/curationis.v42i1.1812

**Published:** 2019-05-23

**Authors:** Stella Kamphinda, Evelyn B. Chilemba

**Affiliations:** 1 Nurses and Midwives Council of Malawi, Lilongwe, Malawi; 2 Faculty of Nursing, Kamuzu College of Nursing, University of Malawi, Zomba, Malawi

**Keywords:** clinical learning environment, clinical supervision, clinical learning, undergraduate nursing students, Malawi

## Abstract

**Background:**

The nurse educators’ role in clinical learning is to define the necessary prerequisites of an ideal clinical learning environment.

**Objectives:**

The purpose of the study was to explore and describe the Kamuzu College of Nursing (KCN) undergraduate nursing students’ perspectives on clinical supervision and support in their clinical learning environment and their preferences in the clinical learning environment.

**Method:**

A mixed method research approach was used to explore and describe clinical supervision from the students’ perspectives on the features of their actual and preferred clinical learning environment. The study’s population comprised all third- and fourth-year undergraduate nursing students (*n* = 219). A sample (*n* = 125) was randomly selected from the population for the quantitative survey of which 120 questionnaires (96%) were valid for analysis. The data collection for qualitative arm of the study comprised interviews conducted through purposive sampling interviewing 20 participants. Survey results were analysed using the Statistical Package for the Social Science (Version 16) and the qualitative data were analysed using the content analysis approach where themes were generated.

**Results:**

The study found that the participants were not satisfied with clinical supervision and support during clinical learning. The participants preferred improved clinical supervision and support in their clinical learning. Comparing the difference between actual and the preferred items of supervision the results were statistically significant at *p* < 0.05.

**Conclusion:**

There is a need to improve students’ clinical supervision and support at KCN. Nurse educators need to plan for clinical supervision and support effectively to promote proficient nursing graduates.

## Background

The nurse educators’ role in clinical learning is to define the necessary prerequisites of an ideal clinical learning environment (CLE). Clinical supervision and support of learners in the CLE forms an optimal clinical learning experience as learners should be satisfied with aspects of personalisation in clinical learning (Berntsen & Bjork 2010:21). Students’ optimal clinical learning is a complex endeavour requiring focused clinical supervision and support and is positively related to levels of cohesiveness, satisfaction and task orientation in the clinical setting (Chan 2002:74). At Kamuzu College of Nursing (KCN) in Malawi, the CLE for the undergraduate nurses has changed since the introduction of the Bachelor of Science in Nursing (BSN) programme in 1996, in terms of low staffing levels, material poverty, increased student numbers and the changing disease profiles amidst the increased student intakes. Anecdotal reports and observations from stakeholders in Malawi reflected concerns about the CLE in terms of quality of supervision and the support that undergraduate nurses received. The impact of the changes in the CLE has not been analysed in terms of sound student learning and what students perceive to promote their learning in their CLE.

The CLE for undergraduate nurses is a multidimensional entity with a complex social context which offers opportunities for students’ clinical learning (Chan 2002:69). These multidimensional entities in CLE comprise the essential elements of social climate, learning opportunities, peer-level interactions, high degree of staff support and morale, interpersonal relationships and feedback, guidance and good interpersonal communication (Chan 2002:70; Cheraghi, Salasli & Ahmadi 2008:30–31). Thus, students’ quality clinical learning relies on these multidimensional entities and understanding on how the CLE influences clinical learning is core to effective clinical teaching.

The paucity of evidence on what promotes the undergraduate nurses’ clinical learning in the CLE in Malawi is an issue needing to be explored because of the stakeholder observations and concerns on the level of clinical performance of the graduate nurses. Supervision is a process of professional support in clinical learning where regular discussions on subject content and visits are made by the experienced and knowledgeable professionals, to socialise the neophytes in their professional roles (Franklin 2013:35). Thus, supervision is key in the CLE for undergraduate nurses as Saarikoski, Isoaho and Leino-Kilpi (2007:1236) attest to its pedagogical and social role dimensions in supporting clinical learning. In support of the notion is a study by Myall, Levette-Jones and Lathlean (2008:1835), which suggested that the allocation of a designated mentor was important to most students in their study. To this end, supervision creates support for a social climate favourable for meaningful lifelong learning in practice settings.

Furthermore, it is desired that the CLE promote supervision among learners for the acquisition of core professional values through sound peer interactions and high degree of staff support and morale. The peer interactions and staff support are crucial in clinical learning as these enhance the development of self-esteem, self-confidence and motivation. Razaee and Ebrahimi (2012:65) in their study found that effective supervision was key to positive CLE because the CLE offered student nurses opportunities for translating the classroom learning experiences into competences and skills necessary for the acquisition of the required competencies. Elcigil and Sari (2011:242) allude to the fact that the support in clinical learning that is demanded by learners is in the form of assessing, teaching and preparatory behaviour. This notion then supports the facets of supervision. No research has been conducted to illuminate on what is an ideal CLE for clinical learning in the Malawian context despite the increased numbers of student intakes, staff shortages and material poverty.

However, Sundler et al. (2014:3) conducted a cross-sectional study with comparative design to investigate student nurses’ experiences of their CLE in relation to supervision. The results revealed that the student nurses were not happy with the quality of supervision and support that they received, meaning that these are crucial to clinical learning.

According to social learning theory, learners are reinforced by being praised for modelling certain behaviour under a supportive environment; therefore, understanding the facets of the CLE at KCN in terms of contributing to or detracting from learning is crucial for an optimal CLE (Young et al. 2014:S42). Helmich et al. (2011:732) and Eraut (2004:251) highlight that learning in practice implies a lot of uncertainty and involves many implicit learning processes. Therefore, the study aimed at analysing the undergraduate nursing students’ perspectives of their actual clinical learning environment and their preferred clinical learning environment.

### Problem statement

The development of skills and competences among undergraduate nursing students is a crucial component of clinical education. During their training, nursing students receive as much clinical exposure as possible and educators implement strategies to assist the students to develop competencies. The CLE provides an opportunity for the undergraduate nursing student to develop the required skills and competences. Strategies in place to ensure students develop the competences despite the increased student numbers and material poverty include recruitment of preceptors or clinical instructors.

Despite these efforts by the college, anecdotal reports and observations reflect stakeholders’ query on the level of clinical performance among the KCN undergraduate nurses. These observations could be related to issues surrounding the CLE such as the supervision and support that is provided. Thus, does the supervision and support provided in the clinical learning promote students learning? Questions arise whether the CLE for undergraduate nursing students has served to equip these undergraduate nursing students more appropriately with the skills required and competences.

There may be a gap between the expectations and reality of the CLE for the students. No studies have been conducted to evaluate the clinical learning environment for the KCN undergraduate nursing students. It was imperative then that this study should be conducted to assess the students’ preferences on their CLE and subsequently improve the CLE for KCN undergraduate nursing students.

### Research purpose

The purpose of the study was to explore and describe undergraduate nursing students’ perspectives of their actual and preferred clinical learning environment.

### Research objectives

The objectives of the study were to:

- explore the undergraduate nursing students’ perspectives on supervision and support in the CLE at KCN
- describe and compare the preferred and actual CLE among undergraduate nursing students.

## Research methods and design

### Research design

The study combined quantitative and qualitative approaches in a concurrent explorative descriptive mixed methods design, underpinned by a pragmatist research paradigm (Creswell 2008; Creswell et al. 2003). The concurrent exploratory descriptive mixed method design was attained by collecting quantitative data concurrently with the qualitative data to enhance the findings. Mixed method research approach was chosen because the breadth and depth of the findings from this approach (Johnson, Onwuegbuzie & Turner 2007:122) were believed to provide a unique understanding of the truth about the CLE for the undergraduate nursing students’ perspectives. This approach also enhanced the descriptions from the undergraduate nursing students’ perspectives on the features of their actual and preferred CLE. Creswell (2008:552) stresses that merging, integrating and linking the two strands of data offers a better understanding of issues. Further, LoBiondo-Wood and Haber (2006) assert that combining methods adds depth and breadth to the results. The results presented here were obtained concurrently from the quantitative and qualitative data. The quantitative part was meant to yield specific results that could be statistically analysed. Then the results were complemented by the actual words of the students from the unstructured questions on the in-depth interviews that adequately provided detailed information about their clinical learning environment experiences. For this study, the epistemology framework and contextualisation of the CLE determined the specific understanding of the research phenomena.

### Study setting

The study was conducted in Lilongwe District in the central region of Malawi, specifically at KCN. Kamuzu College of Nursing utilises Kamuzu Central Hospital (KCH) as its clinical site and is a referral site for all district hospitals in central and northern regions of Malawi. The hospital offers tertiary services to patients plus maternal and neonatal services. The hospital has 1000 beds, with daily bed occupancy of 961 patients. At the time of this study, there were 74 registered nurses. The college was chosen because it is the first college in Malawi to train BSN graduates.

### Study population

The study population comprised all third- and fourth-year undergraduate nursing students (*n* = 219). Polit and Hungler (2004) point that a population should always comprise the entire aggregate of elements in which the researcher is interested. Therefore, the third- and fourth-year nursing students were chosen to participate because they were senior students who by then were expected to isolate learning issues affecting their learning in their CLE. In addition, these were believed to have been allocated to their CLE more than once.

### Study sample and sampling method

A random stratified sample of students was selected from the third- and fourth-year undergraduate nursing students (*n* = 219) (Polit & Beck 2008). Using a table of random numbers, the names of individuals were selected from the population until a minimum of 125 students was obtained. A sample size of *n* = 125 students was calculated based on the assumptions of 75% student satisfaction level at 95% confidence interval and a 5% standard error. Then a finite population factor was used because the target population was small. Purposive intensity sampling method was used for the qualitative data and 20 participants were interviewed.

### Inclusion and exclusion criteria

The inclusion criteria were as follows: being a third-year or fourth-year student with adequate experience with their CLE (Polit & Beck 2008). These students were included because they had long and broad clinical exposure providing nursing care to patients in medical, surgical, paediatrics, obstetrics and gynaecology wards.

### Pilot study

A pilot study was conducted on 11 nursing students at Daeyang College of Nursing to refine the methodology (Burns & Grove 2007). Daeyang College of Nursing trains registered nurses and uses the same KCH as their clinical learning environment. This pilot study was conducted to ensure reliability and validity of the data collection instrument as well as examining if the instruments were reliable to gather the expected information (Polit & Beck 2008). Piloting of the questionnaire also assisted in identifying difficulties and misinterpretations which the participants had with some parts of the instruments. In addition, the pilot assisted in checking the feasibility of the study in terms of resources, time and the willingness of the participants to take part in the study (Polit & Hungler 1999). Following the pilot study, some adjustments were done to refine the research instrument by adding the demographic data which were previously missing and demarcating the actual and preferred items on the questionnaire.

### Reliability of data collection tools

A structured questionnaire developed guided by others’ ideas through the literature review was used to collect quantitative data. The questionnaire consisted of a total of 34 items that were developed based on the issues that contribute to the CLE features. The importance of each item was rated on a 3-point, Likert-type scale ranging with the alternatives of 3 – agree, 2 – neither disagree nor agree and 1 – disagree. The questionnaire comprised summated scales to probe underlying constructs that the researcher wanted to measure. Supervision and support was one of the constructs that was included in the questionnaire. The construct had four items that were identical in the actual and preferred versions (Chan 2002:73). Scale reliability for both versions was confirmed with Cronbach’s alpha coefficients (Chan 2002:73). The coefficient alpha was used to test for internal consistency of scores on the questionnaire because the items were scored as continuous variables (Creswell 2008). Cronbach’s alpha coefficient was symbolised as a continuum with value from 0 to 1; 0 indicated no relationship and the closer to 1 the coefficient was, the more reliable the tool (LoBiondo-Wood & Haber 2006). Literature shows that a Cronbach’s alpha coefficient of 0.70 is acceptable (LoBiondo-Wood & Haber 2006; Tavakol & Dennick 2011). The questionnaire showed reasonable reliable index. The physical conditions on the ward scale consisted of five items (*α* = 0.79 for the actual form and *α* = 0.91 for the preferred form).

### Trustworthiness of qualitative data

The rigour for the qualitative component was addressed by ensuring credibility, dependability and transferability. Thus, the credibility of the research tool was achieved by triangulation methods which is part of quantitative data; the qualitative data were collected through written responses as well as in-depth interviews. The triangulation allowed to overcome the weaknesses of each data set by the strength of another set. Dependability was guided by the interview guide that ensured consistency throughout the interview process. Further, dependability was achieved by reporting the research methodology in detail to promote future researchers to properly assess the extent to which proper research practices had been followed. Transferability was addressed by providing sufficient data in the research report so that readers can assess and evaluate the applicability of the data to other context.

### Data collection

Data collection was done over a period of 1 month. A self-administered structured questionnaire was administered to 125 nursing students who participated in the study. Each participant was visited in their respective settings where questionnaires were left for them to fill and return to the researcher at their convenient time within 2 weeks. Each participant was given an information sheet and consent form to carefully read. After reading, each participant was asked to give consent by signing the consent form before they decided to participate. A contact person was chosen from each group of participants and dates were agreed upon for collecting the questionnaires. Concurrently, in-depth interviews were conducted with 20 participants. A quiet room was identified for facilitating the interviews. In order to facilitate a relaxed and conversational atmosphere, each participant was given a time slot to come to the venue. Explanations were given on the conduct of the interviews. Consent forms were signed before the interviews to affirm willingness to participate and as a requirement in ethical conduct of research. Every effort was made to closely follow the content and meaning of both verbal and nonverbal conversation during the interviews. Probes were used where more information was sought. Sensitivity to the uniqueness of each participant during and throughout the interviews was observed and all interviews were audio-taped for permanent full recording. The interviews lasted for 16 min on average.

### Data management and analysis

Data were prepared for analysis by performing checks to ensure that they were consistent and correct (Polit & Hungler 1999). A database was created using Statistical Package for Social Sciences (SPSS) version 16 and the data were carefully entered. Each category of a variable was given a number (coded) which ranged from 1 to 3. Frequency tables for each variable were generated to locate possible errors by examining the distribution of responses to each item in the data set.

Data were analysed using SPSS where descriptive statistics were computed and these included mean, frequencies and percentages that were calculated to describe the nursing students’ perspectives. Mean scores for each version of each scale and the difference between the mean scores of the preferred and actual versions were calculated to describe the nursing students’ perspectives on the physical environment of their CLE (Alraja 2011:39). Analysis on the differences in undergraduate nursing students’ perspectives of their actual CLE and their preferred CLE was done using a *t*-test (Alraja 2011:39). Literature shows that the *t*-test is used to test for differences between means measured on paired samples. Therefore, in this study, the *t*-test was performed to find out if the mean scores for the actual CLE were more different from the students’ preferred CLE (LoBiondo-Wood & Haber 2006). Hence, the group means for the actual version and the preferred version were used to produce a value for *t* (Midgley 2006:342). The qualitative data were analysed using content analysis where themes were created and the results were integrated in the discussions.

### Ethical considerations

Approval of the research proposal was obtained from College of Medicine Research Committee (permit number P. 08/13/1453). Permission to conduct the study was sought from Principal KCN in written letter form for formality. Consent was obtained from the subjects to indicate their willingness to participate in the study. Each participant was asked to read the client information sheet in order to be informed about the purpose of the research, selection method, data collection procedure and possible risks and benefits. The subjects were assured that participation was voluntary and that they could withdraw from the study any time they like. Confidentiality and anonymity were guaranteed by ensuring that data obtained will not be shared with anyone other than the researcher (LoBiondo-Wood & Harber 2006).

## Results

A total of 125 students participated in the study with 120 questionnaires valid for analysis, providing a response rate of 96%.

The majority of the participants 92% (*n* = 110) were in the age group of 21–29 years, with mean age of 25 years. Fewer participants (4%, *n* = 5) were in the age group of 30–39 years and the remaining 4% (*n* = 5) were in the age group of 20 years and below.

In terms of gender, the majority of the participants (76%, *n* = 91) were females and 24% (*n* = 29) were males, reflecting the issue of gender diversification. Fifty-nine per cent of participants (*n* = 71) were in their third year of study and 41% (*n* = 49) were in their fourth year of study.

The majority of the participants (98%, *n* = 118) had been allocated to 3–5 wards. Table 1 presents the detailed demographic characteristics of the participants.

**TABLE 1:** Demographic characteristics of participants.

Characteristics	Frequency	Percentage
**Age group**		
21–29 years	110	92
30–39 years	5	4
20 years and below	5	4
**Gender**		
Female	91	76
Male	29	24
**Year of study**		
Year 3	71	59
Year 4	49	41
**Number of allocations to the clinical learning environment**		
3–5 wards	118	98
1–2 wards	2	2

The results indicate that 85% (*n* = 102) of the participants had found 1–3 registered nurses (RNs) in their wards, while 14% (*n* = 17) reported to have found 4–6 RNs in the wards and only 1% (*n* = 1) reported to have found 7–10 RNs in their wards.

The duration that the students were given to remain in one ward varied. The results reflected the majority (86%, *n* = 103) of the participants indicated that their time period per allocation on average was 4–7 weeks. Fewer participants (9%, *n* = 11) indicated that they had 1–3 weeks of placements, while 5% (*n* = 6) indicated that their allocation on average lasted for 8–10 weeks. The results further showed that the participants’ satisfaction with the number of weeks they were allocated to also for an experience in a specific ward varied.

The results showed that out of the 86% (*n* = 103) who indicated 4–7 weeks of clinical placement, only 48% (*n* = 49) indicated that the 4–7 weeks period was adequate for learning, while 34% (*n* = 35) of the participants indicated that this period was not adequate for learning and only 3% (*n* = 3) said that the period was too long for learning. Table 2 summarises the results.

**TABLE 2:** Demographic characteristics of the clinical learning environment.

Characteristics	Frequency	Percentage
**Number of registered nurse per ward**		
1–3 registered nurses	102	85
4–6 registered nurses	17	14
7–10 registered nurses	1	1
**Number of weeks per allocation**		
4–7 weeks	103	86
1–3 weeks	11	9
8–10 weeks	6	5
**Adequacy of period of each allocation**		
Adequate for learning	67	56
Not adequate for learning	47	39
Too much for learning	6	5

### Comparison of the existing supervision and support received with what the students preferred

The mean scores for the actual version of the items in this scale ranged from 1.63 to 1.88. The preferred version’s items mean scores ranged from 2.82 to 2.90; the participants had preference in terms of supervision and support in clinical practice from staff nurses and lecturers. A paired sample’s *t*-test showed that the mean scores between the actual and the preferred forms were statistically significant at *p* < 0.05, suggesting that the participants did not receive feedback from their supervisors, the registered nurses were not interested in teaching the students, the students were not satisfied with the supervision they received during their practicum and they did not feel they received individual supervision (see Table 3 for details).

**TABLE 3:** Mean score differences between the actual and preferred versions for support and supervision scale.

Item	Scores				Mean difference	*t*	95% CI of the difference		df	Sig. (2- tailed)
	Actual		Preferred							
					Lower				Upper
	M	SD	M	SD					
I continuously received feedback from my supervisor	1.70	0.816	2.89	0.406	1.19	−14.49	−1.355	−1.029	119	0.000
The registered nurses were interested in teaching me	1.88	0.735	2.90	0.328	1.02	−14.52	−1.155	−0.878	119	0.000
I am satisfied with the supervision I received during my practicum	1.69	0.708	2.84	0.430	1.15	−15.84	−1.294	−1.006	119	0.000
I feel I received individual supervision	1.63	0.723	2.82	0.467	1.19	−15.30	−1.346	−1.037	119	0.000

CI, confidence interval; M, mean; SD, standard deviation; df, degrees of freedom.

### Qualitative results

The qualitative data were analysed in four major themes.

#### Theme 1: Availability of a multidisciplinary personnel

All participants talked on the availability of multidisciplinary personnel in all the wards they had placements. Some participants reported that their CLE was conducive because the personnel were interested in teaching them. This made the participants feel accepted in the CLE.

One participant had this to say:

‘There are specialists there in the CLE who can teach you thoroughly whenever you want to learn.’ (Participant 6, female, year 3, aged 22)

However, the participants alluded to the fact that this was only observed from the doctors. Two participants narrated as follows:

‘Those people who are interested to teach us are doctors and not fellow nurses, doctors teach a lot at KCH not the nurses.’ (Participant 11, female, year 3, aged 24)

‘I was in a certain ward where the incharge was not willing to teach us, you ask something she could show lack of interest.’ (Participant 11, female, year 3, aged 24)

#### Theme 2: Shortage of staff nurses in wards

Shortage of staff nurses was another factor that impacted the positive aspect of the CLE at KCH. The participants argued that the availability of sufficient staff was an important factor for creating a positive CLE. They confirmed that the shortage of staff nurses affected the services and support they had received.

The rationale provided included:

‘The nursing staff is not adequate … as a result nurses are most of the times busy doing ward activities than teaching students.’ (Participant 4, female, year 3, aged 26)

The participants stated they were not happy practising on their own during clinical learning. They indicated that students were left to practise on their own with no support and guidance from staff nurses or lecturers.

The rationale provided was:

‘At least they should appreciate that these are students, they come here to learn so they should teach students rather than just leave them on their own.’ (Participant 10, female, year 3, aged 20)

The participants indicated that support, guidance and supervision were more desired by nursing students while in their CLE. However, the participants stated the students preferred more of the support from their faculty members of staff than from ward nurses.

The following excerpt illustrates the point:

‘We lacked much supervision from our lecturers, we are supposed to be supervised mostly during our clinical placements but you find out that we spend about a week without seeing supervisors. … You find a supervisor visit once a week or not even a single visit in a week.’ (Participant 1, female, year 4, aged 30)

The participants stated that the absence of proper supervision system exposed them to numerous scenarios that made them to compromise their learning, as one participant’s excerpt illustrates:

The lectures were not there, we were finishing the whole allocation without being supervised by the lecturers so sometimes we were copying bad things from the nurses because lecturers were not available for us. (Participant 6, female, year 3, aged 22)

Despite that the students were not supervised, the participants indicated that they were used as a pair of hands, as one participant stated:

When the supervisor is not coming you are still used to cover shortage by the ward incharge. (Participants 5, male, year 4, aged 27)

The proximity of the CLE to the college gave the participants hope of having a very powerful CLE. On the contrary, the participants found themselves less motivated because of the scarcity of their lecturers.

One excerpt illustrates:

Just because KCH is near KCN we expected supervisors to be visiting from now and then, but this is not the case. (Participant 1, female, year 4, aged 30)

#### Theme 3: Availability of learning resources

The participants indicated that their CLE had rich learning experiences to provide adequate learning experiences. Thus, the CLE had sufficient numbers of patients with a variety of health problems as illustrated by one participant:

KCH is a good learning environment for students, it is a place where students can get all what they desire to learn. I mean in terms of objectives as required by their courses …. and there are so many conditions. (Participant 8, Female, year 3, aged 28)

However, most participants affirmed that despite the adequate numbers of conditions in the CLE there were inadequate material resources and as a result it did not support their learning effectively. Some of the rationales provided to this were:

We learn the ideal things but when we go to the clinical area we fail to do the ideal because of the resources, talk of gloves, drapes, aprons. (Participant 3, male, year 4, aged 26)

Sometimes with scarcity of resources, when there is a client to be assisted, you find the sister (nurse) telling you just leave the patient we do not have resources. (Participant 11, female, year 3, aged 24)

The inadequate resources failed to support the learning among the participants as they reported that there was a lot of improvising.

The excerpt illustrates:

Inadequate resources, this makes us to know much about improvising other than doing the actual thing, hence we tend to forget the most crucial areas when providing care. (Participant 3, male, year 4, aged 26)

#### Theme 4: Poor nurse–student relationship

The participants also alluded to the poor nurse–student relationship as a barrier to the support to learning. They indicated that the poor nurse–student relationships failed to promote a conducive CLE for promoting students’ learning.

Some participants had this to say:

When it comes to learning they should consider that we have our own objectives, they expect us to do the same tasks which they are supposed to do, and when you say I cannot do this they regard you as being rude. (Participant 1, female, year 4, aged 30)

The way we were treated at one ward, the nurses could say some other things about us then we could feel out of place. Like here you cannot learn and that hindered us to learn effectively. (Participant 4, female, year 3, aged 24)

## Discussion

The results of the study show that the participants were not satisfied with the clinical supervision and support that was rendered during their clinical learning. It is important that KCN educators consider the CLE of their undergraduate nurses by identifying an optimal CLE from their students’ perspectives to promote learning (Young et al. 2014:542). A supportive culture in CLE is a cornerstone for effective clinical learning. Schon (1983) described the clinical area as a swampy area; if participants had preference to the CLE in terms of supervision and support, this preference would have been considered in line with their learning goals and value orientations (Chan 2002:71).

The participants had preferred improved clinical supervision and support, which affirms the notion that supervision creates a social climate for support in clinical learning effectiveness. Preference has been determined through the high total mean scores on the items in Table 3. These participants were senior students who were expected to have had enough exposure with their CLE, and 86% (*n* = 103) had more than 4–7 weeks of clinical placements. This factor is very important in determining the functionality of the KCN CLE.

Chan (2002:71) points out that comparing the actual and preferred CLE offers opportunities for understanding a setting better with insight into the existing problem areas. This is because the theoretical framework for clinical learning environment studies alludes to the fact that students’ learning is positively related to the levels of cohesiveness, satisfaction and task orientation, and negatively related to levels of friction and disorganisation (Chan 2004; Cheraghi et al. 2008).

All the participants (100%) had been allocated to the CLE for more than once; the participants were exposed to a number of clinical settings where they alluded to have had rich learning experiences. However, despite the number of allocations and rich learning opportunities, 85% of the participants indicated there were few RNs on duty during their allocation, as depicted in Table 1. This result reflects the dissonance in the quality of clinical supervision and support that the participants received; thus, there was low degree level of staff support and morale to promote clinical learning. The social climate was rather quiet with minimal student–nurse interactions; then students’ learning might have been suppressed and may have influenced the quality of learning negatively.

A social culture in this CLE of decreased peer-level interactions existed because of the staff shortages and absence of nurse educators. The study results further reveal that the undergraduate nursing students received inadequate support and supervision while in the CLE (*p* < 0.05). In such situations, the students may not see the ward nurses as supervisors but rather as colleagues and the ward nurses may also forget or ignore their supervisory responsibility such that students may be providing patient care without being supervised and learning (Warne et al. 2010:810). In the qualitative data, the participants attest to the inadequate support from the qualified nurses in the wards; this was because of poor nurse–student relationships, the unwillingness of staff nurses to teach and inadequate resources. The multidimensional elements of the CLE emphasise good interpersonal communication, high degree of staff support and morale. However, it can be concluded that the KCN CLE lacked supervision and support entities to be an ideal CLE as perceived by the participants.

Babenko-Mould et al. (2012:223) assert that there is evidence for the fact that students take note of the staff nurses’ professional behaviours in practice. Similarly, Bergjan and Hertel (2013:4) evaluated students’ perception of their clinical placements in Germany and found that 54% of the students had unsuccessful supervisory experiences that impacted learning. In the same vein, Warne et al. (2010:813) reported their study where 47% of the students had unsuccessful supervisory experiences. Further, in a study by Kachiwala (2007:47), 32.95% of the participants experienced unsuccessful supervision and support in the clinical placements at Malamulo in Malawi. The inadequate staff ratios in the CLE influenced learning negatively according to the participants’ excerpts in the qualitative data; if nursing colleges are to train for quality and safety outcomes, then support and clinical supervision in the CLE is crucial. This is because support and clinical supervision promotes the learning climate that fosters increased peer-level interactions (Berntsen et al. 2010:17). Thus, staff nurses’ support to students learning in their CLE results in empowerment and self-efficacy among learners (Babenko-Mould et al. 2012:223; Saarikoski et al. 2007).

However, clinical learning requires that educators and staff nurses provide feedback to the nursing students and this is not possible in settings where staff shortages are present like at KCN. It was evident that from the qualitative data excerpts that the participants did not receive feedback from their supervisors; thus, it was reported that the registered nurses were not interested in teaching the students and that students were not satisfied with the supervision they received. The differences for the two items were statistically different (*t*-value -14.49 and -15.30, respectively). Learning in clinical environment is by doing; hence, supervision and support from the ward nurses and faculty is essential (Papp, Markkanen & Bonsdorff 2003:265). Feedback offers learners an interactive process that aims at providing insight into learning performance (Clynes & Raftery 2008). The absence of feedback then confirms the lack of support and clinical supervision received by the undergraduate nurses in the CLE. The participants would not have expressed satisfaction with their CLE because of the missing essential elements in the multidimensional entities of the CLE despite the rich learning opportunities in the CLE.

Furthermore, the participants had reported shortage of resources and staff nurses not willing to teach them in the CLE; these are the factors that might have failed to make the CLE optimal for clinical learning and hence reduced the participants’ preference for the CLE. Nurse educators in Malawi need to be cognisant of the required supervision and support in the CLE to create learning opportunities for the undergraduate nursing students to promote clinical learning. Saarikoski, Leino-Kilpi and Warne (2002) cite the results of their comparative study between Finnish and British nursing students that revealed that a better system of supervision by clinical teachers was interpreted as a major reason for students’ higher satisfaction with their clinical learning, particularly with the Finnish students. Supervision involves the day-to-day interactions with the student nurses of varying amounts of formality between a lecturer or ward nurse and a student with the responsibility for promoting the achievement of learning objectives. The results showed that the participants did not continuously receive feedback from their supervisors (mean difference = 1.19).

Nurse educators need to structure the CLE to promote opportunities among the students to link theory and practice through effective feedback mechanisms. This study result is related to a study finding by Cahill (1996), where students indicated that they rarely received praise from their supervisors. Similarly, Raftery (2001) asserted that students are only informed of inaccuracies at the end of a placement when room for improvement is not available. The lack of feedback can lead to decreased levels of student self-worth which may have a negative impact on succeeding practice placements. This is because the CLE has to accord the students opportunities to perform nursing care and practise nursing skills while connecting performance with learning (Berntsen & Bjork 2010:18). The results further showed that the students wanted to be supervised on a regular basis. This matches well with the results by Nylund and Lindholm (1999:281) where students wished the supervisor to be in the background whenever they need them. In their suggestion, Saarikoski et al. (2007) stated that supervision is supposed to be done on a regular basis to identify solutions to problems and improve performance.

This is a confirmation that nursing students need a secure base from which to explore and learn (Sundler et al. 2014). They need encouragement both when they perform well and when they perform unsatisfactorily. Elcigil and Sarı (2011:495) asserted that positive feedback gives students occasion to reflect on their own development. Therefore, the need for the students’ supervision development cannot be diluted because supervision provides room for feedback to the students.

The participants indicated that the ward nurses expected them to do the same tasks which the nurses were supposed to do. This entails letting the students perform on their own as any other qualified nurse does. Without supervision there is no room for feedback and without feedback students’ mistakes go uncorrected and good performance is not reinforced. Walsh (2010) and Clynes and Raftery (2008) point out that providing feedback is a vital aspect of supporting a student in the CLE and is part of the learning process. The qualitative results of this study showed that supervisors’ visits to the wards were scanty despite the fact that the CLE is in the vicinity. The participants further reported that the ward nurses were not interested in teaching them and their lecturers visited the ward to check if the students were in the ward and not to teach them. This is in line with what Cahill (1996) found that supervisors usually were for fault finding. It is difficult for a supervisor who does not work with students to give objective feedback as they may lack points of reference.

However, sound pedagogical principles need to guide the clinical supervision approach at KCN to facilitate positive impacts on clinical learning outcomes. The majority of the participants had perceived that they missed supervisory relationship mostly with their lecturers than the ward nurses. This study finding is in agreement with the finding of Cheraghi et al. (2008:30) where factors influencing the clinical preparation of BSN nursing students were studied in Iran and found that students lacked educators for guidance and had experienced unsupportive and non-scientific relationship among the staff nurses. The participants in this study viewed the poor nurse–student relationships in the qualitative data as barriers to meaningful learning in their CLE.

To this end Heshmati-Nabavi and Vanaki (2010) identified that nurse educators were a source of support for nursing students in Iran, affirming the fact that good interpersonal relationships with educators in clinical learning is a catalyst to effective learning. Furthermore, in this study the nursing students’ experiences reflected that there was no human relationship in their CLE because of the lack of interest that the staff nurses had shown. Literature postulates that in a situation where there is inadequate supervision, lecturers fail to understand the opportunities students receive from their CLE and how the students make use of them (Mabuda, Potgieter & Alberts 2008:1838). Despite the rich learning experiences available in the CLE at KCN, the participants had a preferred CLE.

It is also important to note that through nurse educators’ support, the students gain awareness of the learning situation by reflecting over their own practice. Shen and Spouse (2007:329) suggested that students doing their clinical learning favoured proper supervision which had a humanistic approach as this helped them to develop into proficiency. The participants in this study affirm the situations where the staff nurses were feared by the student nurses in their CLE. There is a need for KCN educators to have a strong partnership with the staff nurses by developing clear expectations on both sides so that students can be helped to become skilled and fit for practice at their level as RNs. Myall et al. (2008:1837) alluded to the fact that most students valued the allocation of a designated mentor because the mentor had offered them opportunities for feedback and learning.

However, designation of a mentor may not be without challenges in the Malawian context because of the shortage of nurses in posts in most public hospitals. In a study by Jokelainen et al. (2013), mentors lamented of lack of support from colleagues in the ward as well as from educators. The mentors further preferred regular visits of educators to CLE and their involvement by working alongside students. However, a study by Sundler et al. (2014:3) revealed that students with their own personal preceptors reported to have had a more positive experience of the supervisory relationship than did students placed in a patient room with a different preceptor from shift to shift, which emphasises an organised system of supervision (Pillay & Mtshali 2008). Similar to mentorship, preceptors in Canada found it difficult to cope with the demands of their roles in clinical teaching (Mantzorou 2004); then there is a need for the KCN educators to deploy a supervision and support model that will favour their CLE setup to enhance meaningful learning among the undergraduate nursing students. In the same notion, a study by Lillibridge (2007:49) revealed that preceptors also expected support from the nursing faculty in socialising the student nurses. This leads to a conclusion that both mentors and preceptors still need support from faculty members in the CLE for promoting learning (O’Connor 2006).

The results further indicated that the participants wished to be treated as individuals. The statistical test (*t*-value -15.30) on this item was more extreme than the critical value, indicating that the students were not satisfied with the interpersonal relationship that they received. This result is congruent to the finding by Nylund and Lindholm (1999:284) where students also expressed a wish to be treated as individuals. Students who are treated as individuals in the CLE feel welcomed and they can easily fit into the health care team. High degree of staff support and morale is key to learning motivation in clinical settings. Hence, they can ask questions where they are not competent and they would be coached efficiently. A clinical learning environment that is supportive helps students to reach their potential and offers the students an inspiration for success. This is in line with humanistic theories of learning, andragogy theory and critical pedagogy theory that emphasise the need for increasing autonomy and giving a high priority to satisfying learners’ needs. Therefore, with adequate supervision, the students will have feelings of self-worth and autonomy which are important factors in making them deeply engaged in clinical learning.

### Limitations of the study

The study site was not wholly representing most of the sites where undergraduate nurses are placed for clinical learning. As such it is noted that the results of this study may not be generalised to other areas in Malawi. There was need to assess how the undergraduate nurses attained their required learning outcomes in the CLE in the absence of supervision and support to check on how learning was achieved.

### Recommendations

The study has provided insight into the social context of the CLE at KCN regarding how it impacts learning and how learners view their CLE. To promote clinical learning among the undergraduate nurses in the clinical settings, it is recommended that the challenges that are currently in the CLE should be addressed in line with the curriculum design. A proper supervisory model has to be instituted to enhance the role of nurse educators. The roles of the nurse educators in clinical teaching need to be explicitly linked to their professional roles to ensure that they avail themselves to the ward settings when students are in the clinical area. There should be involvement of policymakers in negotiating for the establishment of RN posts in the various wards to increase the number of staff nurses in posts.

## Conclusion

Supervision creates a social climate for support in clinical learning attested by the participants and has to be promoted by all nurse educators. The participants in this study had a preferred CLE which has multidimensional entities, that is, regular supervision and support by nurse educators and staff nurses. The findings of this study reveal that the changes in the CLE have impacted clinical learning among the undergraduate nurses. Despite the participants’ wanting to be supervised on a regular basis, there were minimal contacts with their lecturers, which is the most important factor that influenced the participants’ perception of their CLE. Regardless of the allocation times for clinical placements of 4–7 weeks, some participants indicated the time was not adequate, implying that the participants failed to focus on clinical learning. There was no supervision feedback offered among the students despite that more supervision feedback was desired by the participants in the CLE. There has been minimal support from staff nurses, which also influenced the participants’ perception of the CLE. Nurse educators need to increase their visits to the CLE to increase student motivation and ensure that the student’s learning opportunities in the CLE are rich despite the social climate.
